# Open questions in chemical glycobiology

**DOI:** 10.1038/s42004-020-00337-6

**Published:** 2020-08-07

**Authors:** Mia I. Zol-Hanlon, Benjamin Schumann

**Affiliations:** 1 The Chemical Glycobiology Laboratory, The Francis Crick Institute, 1 Midland Rd, London NW1 1AT, UK; 2 Department of Chemistry, Imperial College London, 80 Wood Lane, London W12 0BZ, UK

## Abstract

Glycans are ubiquitous in biology, but their complex structure and biosynthesis have challenged research of their wide-ranging roles. Here, the authors comment on current trends on the role of chemical methodologies in the field of glycobiology.

At times in which science has a key role in society, we would do well to remember that interdisciplinarity is a vital ingredient to scientific progress. Science across boundaries has always played a key role in the glycosciences. Chemical methodologies in particular have been indispensable even since the 1920s, when antigenic carbohydrate-protein conjugates were generated using diazonium salts of bacterial glycans by Avery^1^.

A considerable proportion of all proteins are glycosylated^2^, and carbohydrates (glycans) decorate the surface of every living cell. The chemical structure of monosaccharides allows glycosidic bonds to vary in their regio- and stereoisomerism. With the added complexity of branching, glycans display a great diversity of shape and thereby function. It is often surprising to those new to the field how much influence glycans can have in biological systems. For instance, many viruses, including HIV and the coronaviruses responsible for ongoing and past outbreaks, “shield” their surface proteins heavily by glycosylation, thereby influencing their vulnerability to the immune system^3,4^. Therefore, target glycosylation has to be a key consideration in vaccine development. There are countless other examples of the importance of glycans in many of the most pressing topics in biology from cancer to immunology, for which we direct the reader to more comprehensive resources to gain further insight^2^. Despite their now clear relevance in many fields in biomedicine, glycans have often been overlooked relative to the other major biopolymers, due to technical roadblocks unique to the glycosciences. These are summarised below.

## Challenges in glycobiology

Glycans are not directly encoded in the genome; they are secondary gene products, built through the combinatorial interplay of glycosyltransferases (GTs) and glycosidases. The repertoire of these enzymes determines the glycome: the identity of glycan structures, free or as part of glycoconjugates, in a given cell/tissue/organism at a given point in time. Particular predictions about the glycome of a cell can be made by following biosynthetic principles and enzyme specificities. This is especially important in bacteria that have a limited set of carbohydrate-active enzymes which can serve as predictors of glycan structure based on genome mining^5^. In eukaryotes, biosynthetic considerations are generally complicated by a much larger enzyme repertoire that displays redundancy and functional compensation. The dynamics of processive glycosylation within the secretory pathway are thus poorly understood.

Glycans cannot easily be mutagenised. Biosynthetic processes of glycans are far more dynamic than those of nucleic acids and proteins that follow a largely linear information flow of primary sequence. While the well-established and routine editing methods for the latter biopolymers have been key to the great leaps in our understanding of them, swapping one monosaccharide for another in the biosynthesis of a native glycan is often all but impossible. This makes many of the experimental paradigms common for determining function of protein and nucleic acids infeasible, necessitating alternate approaches to the study of glycans.

Glycan sequencing is a longstanding bottleneck. Mass spectrometry (MS) is the most widely used method, but is complicated by biological (e.g. microheterogeneity), technical (e.g. difficulty in enrichment), and physical (e.g. ionizability) challenges. Other methods such as nuclear magnetic resonance (NMR) spectroscopy are powerful but typically require larger sample amounts. As the glycans produced by a cell can differ greatly based on cell type, environmental cues, and disease states such as cancer^2^, this difficulty in measuring the state of the glycome is a major hindrance.

**Fig. 1:**
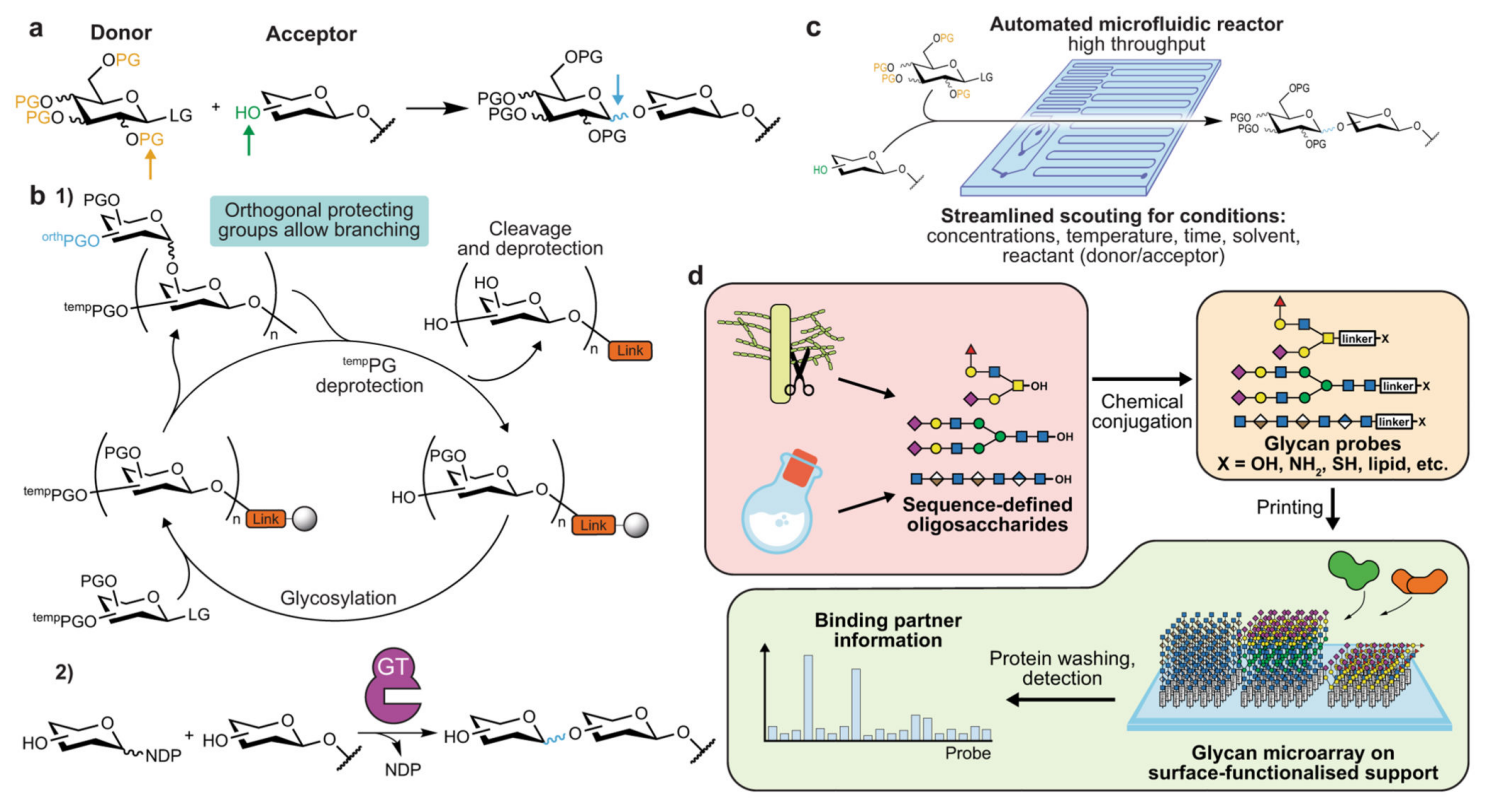
Synthesising and arraying carbohydrates. **a** Multiple selectivities must be managed in the glycosylation reaction: regioselectivity between similarly nucleophilic hydroxyl groups (green), stereoselectivity at the anomeric centre (blue) and branch points influencing protecting group (PG) chemistry (orange) (LG: leaving group). **b** (1) Cyclic, automated glycan synthesis. The accessibility of glycans has been improved by the development of generalised solid and solution-phase syntheses, allowing for streamlined automation of successive reactions and minimisation of the number of steps. Key to this has been protecting group chemistry, including management of orthogonal protecting groups for branching, selection of highly stereoselective reactants and chemistry of the support-bound cleavable linker. (2) Chemoenzymatic glycan synthesis. Nature has evolved a huge array of GTs to add sugar moieties (most commonly using sugar nucleotides as donors, NDP: nucleotide diphosphate) with exquisite selectivity. Efforts to mine genomes for GTs that catalyse otherwise difficult steps have boosted yields and shortened syntheses of biologically relevant glycans. **c** High-throughput glycosylation condition scouting by an automated microfluidic platform for the systematic screening of multiple reaction variables. **d** The glycan microarray technique. Glycans, obtained from natural or synthetic sources, are conjugated to immobilisable groups at their reducing end, such as reactive functionalities or lipids. Thereby, glycans can be spotted on suitable matrices such as nitrocellulose or glass slides in femtomole amounts with an automated printer. Hundreds of glycan probes in parallel can then be screened for binding by proteins using washing and detection, similarly to an ELISA.

The provision of amounts of glycans sufficient for their study is often difficult. While certain glycan structures can be isolated from natural sources in high purity and abundance, this is not the case for most structures of biological importance. The complex, branching structure of many glycans also makes them highly challenging to synthesise, due to the exquisite stereo- and chemoselectivity required, further impeding progress on their investigation (Fig. 1a).

Finally, the identification of glycan-binding partners is challenging. While long polysaccharides can be immobilised for platebased assays, this is not the case for shorter oligosaccharides^6^. Glycans are thus often refractory to standard immunological techniques such as enzyme-linked immunosorbent assays (ELISAs). This often makes assigning specific biological functions to individual glycan structures in complex biological environments an issue.

## Solutions employing chemical biology

Challenges spark creativity. The limited ability of molecular biology methods to tackle the above technical roadblocks has provided an incentive for chemistry to rise to the challenge. Now a key part of the modern glycosciences, chemical glycobiology has become a fertile ground for optimising methods in general; due to the inherent difficulties in the field, methods that work in the glycosciences often prove to be state-of-the-art and transferrable to other fields.

While chemical glycobiology is a tremendously creative area, a small number of methods have particularly pushed the field towards maturity as a discipline within modern biology.

### Carbohydrate chemistry

While the synthesis of carbohydrates is a century-old discipline, the past two decades have seen a revolution in the throughput and accessibility of complex oligosaccharides. This development has been fuelled by innovations in automated glycan synthesis using both solid and solution phases^7,8^, as well as by innovations in chemoenzymatic syntheses^9^ (Fig. 1b). As a result, many more biologically important glycans are now synthetically accessible. Cutting-edge synthetic methods still work best in a chemistry-focused laboratory, due to the elaborate compound purification and characterisation techniques demanded. The efforts of the synthesis community are directed towards expanding the repertoire of accessible structures as well as improving the economy of chemical transformations. While these tasks are deeply rooted in classical synthetic methodology, automated approaches to scouting reaction conditions^10^ (Fig. 1c) and continued discovery of new carbohydrate-active enzymes (http://www.cazy.org) are sure to further advance this subfield.

### Glycan microarrays and display techniques

A chemistry-based solution to the lack of immobilisability of natural glycans was the development of glycan microarrays. Conjugation to immobilisable linkers or to lipids^11,12^ has allowed for parallel robotic printing of glycan probe libraries in miniscule amounts (typically femtomoles) on a solid support. These probes are then screened for interaction with potential binding partners similarly to an ELISA (Fig. 1d). While glycan microarrays have existed for decades, they remain state-of-the-art and have become a key resource in understanding glycan recognition systems. The next years will likely see novel arraying methods come to maturity, such as multiplex bead arrays which aim to boost throughput^13^, cellbased arrays to present glycoconjugates in a near-native environment^14^, and liquid arrays of densely conjugated, DNA-barcoded virions^15^. It is imperative that the scientific community recognises glycans as a major determinant of biological function, and that the need to profile glycan binding is matched by the provision of screening facilities.

### Metabolic oligosaccharide engineering

**Fig. 2:**
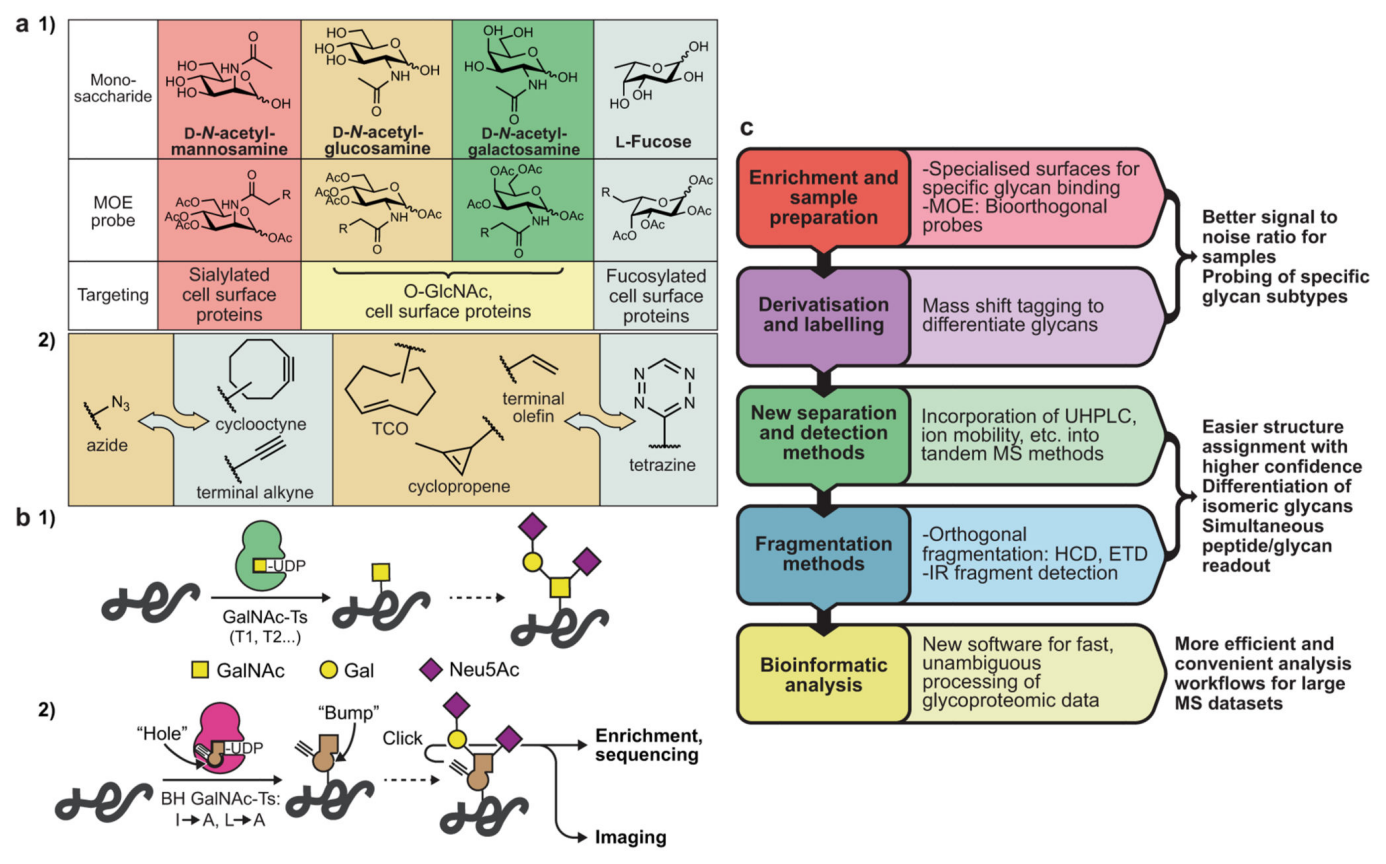
Metabolic oligosaccharide engineering and glycan sequencing. **a** Bioorthogonal chemical probes for metabolic glycan tagging. (1) The ability of some GTs to accept non-natural substrates has allowed for the development of a range of clickable probes enabling enrichment, MS-glycoproteomics and imaging of glycoproteins. (2) A selection of bioorthogonal functionalities. While the copper catalyzed azide-alkyne 1,3-dipolar cycloaddition (CuAAC) and strain-promoted version (SPAAC) (first panel) are the classic examples, another major class involves the inverse electron demand Diels-Alder reaction between tetrazines and alkenes (second panel, TCO: trans-cyclooctene). **b** The “bump-and-hole” strategy for MOE. (1) Humans have approx. 20 isoenzymes of the *N*-acetylgalactosaminyl transferase enzyme family (GalNAc-T1 through T20) that initiate O-glycosylation, with hierarchical and compensatory function complicating their study. (2) Structure-based design of a bump-and-hole (“BH”) double mutant of a single GalNAc-T allows for installation of a gain-of-function MOE reporter system for that enzyme’s activity in cells. **c** A summary of technical advances related to glycan sequencing techniques in recent years. UHPLC: ultra-high performance liquid chromatography, HCD: higher-energy collisional dissociation, ETD: electron transfer dissociation.

In the early 1990s, Reutter observed that certain GTs can accept chemically modified nucleotide-sugars as substrates^16^. Chemists soon set out to equip these glycans with chemical, editable functional groups that can be traced after incorporation into glycans by the cellular biosynthetic machinery^17^. A number of these metabolic olisaccharide engineering (MOE) probes have been developed since; many of these display bioorthogonal click handles to facilitate specific tagging. This technology offers an elegant solution for many of the technical roadblocks discussed above: incorporation of MOE probes allows for enrichment of glycans, and their identification by proteomics and imaging^18^ (Fig. 2a). In order to truly enter modern biology, next-generation MOE techniques must focus on specificity, enabling the development of reporters for subtypes of glycans. One strategy to achieve such specificity is the “bump-and-hole” tactic in which a GT is engineered to accommodate a chemically modified sugar that is ideally not used by wild-type enzymes. This approach yields a bioorthogonal reporter of glycans synthesised within cells by the selected enzyme. We have used this tactic to inform on the cellular substrate specificities of members of the human GalNAc-T GT family^19–21^ (Fig. 2b). Probes with high potency and specificity for cytoplasmic and nuclear O-GlcNAc glycosylation have also been developed, allowing for studying these glycoconjugates by imaging and MS-proteomics^22,23^. The next years will see this and similar approaches to render MOE more specific to more reliably inform on the biological implications of glycans.

### Analytical chemistry

The glycosciences have seen some of the most elaborate technological advances in MS. Along with technical innovations in all areas of the sequencing workflow from sample preparation and enrichment to data analysis, glycan structure determination has become far more tractable in recent years (Fig. 2c). This is especially apparent in MS-glycoproteomics, where the readout of both glycan and peptide sequences has been achieved through orthogonal fragmentation techniques such as higher-energy collisional dissociation (HCD) and electron transfer dissociation (ETD)^24^. Glycopeptides typically require enrichment to increase the signal to background ratio, and bioorthogonal chemistry has provided a means to this end^18^. The use of specialised surfaces for the specific capture of glycans from complex biological mixtures has shown promise to this end^24^. Distinguishing between isomeric monosaccharides as well as different anomeric linkages remains a particular challenge, especially in glycan structures that cannot be inferred by biosynthetic considerations. While great improvements have been made to liquid chromatography in recent years, additional innovations are required. Emerging approaches to dissect glycan structures in unprecedented detail include ion mobility mass spectrometry^25^ and laser-induced infrared spectroscopy^26^ to unravel the molecular identity of each monosaccharide and linkage. The next years will see further implementation of these techniques into routine glycan analysis, allowing the role of glycans to be probed.

## Outlook

Progress on chemical methodologies has greatly expanded our toolbox to dissect the wide-ranging roles of glycans. The coming years are sure to bring more innovative chemical biology-based solutions to the challenges posed by studying glycobiology, providing deeper insights and making the field more accessible.
